# Characterization of a Lab-Scale Process to Produce Whole IgG Antivenom Covering Scorpion Stings by Genus *Tityus* and *Centruroides* of Colombia

**DOI:** 10.3390/ph15091047

**Published:** 2022-08-25

**Authors:** Sebastian Estrada-Gomez, Vitelbina Núñez, Leidy Johana Vargas-Muñoz, Carlos A. Madrid-Bracamonte, Lina Maria Preciado

**Affiliations:** 1 Grupo de Toxinología y Alternativas Terapeuticas, Facultad de Ciencias Farmacéuticas y Alimentarias, Universidad de Antioquia UdeA, Medellin 050010, Colombia; 2 Tech Life Saving (TLS), Tech Innovation Group Company, Medellin 050022, Colombia; 3 Escuela de Microbiología, Universidad de Antioquia, Medellín 050010, Colombia; 4 Facultad de Medicina, Universidad Cooperativa de Colombia, Medellin 050012, Colombia

**Keywords:** scorpion, venoms, LD_50_, antivenoms, IgG, Colombia

## Abstract

Scorpion stings are a public health event in Colombia lacking official epidemiological data, and are considered a medical emergency. Despite the two local producers of antivenoms, neither of them is currently manufacturing scorpion antivenoms. We present the characterization of a lab-scale process to produce the first specific scorpion antivenom for Colombia, formulated to cover scorpion stings produced by *Tityus pachyurus*, *Tityus asthenes*, *Tityus fuhrmanii*, *Centruroides* spp. To do so, rabbits were immunized by subcutaneous injection with each venom using an immunization program of 3 months. After each rabbit reached the required IgG concentration, rabbits were bled, and plasma was separated by decantation under refrigeration. Immunoglobulins were purified from each hyperimmune plasma using a methodology including precipitation with ammonium sulfate, thermocoagulation, and purification through an ultrafiltration process using a ready-to-use and reusable laboratory crossflow tangential cassette with a polyethersulfone membrane. Each hyperimmune plasma was processed by being separated and freeze-dried at the end of the process. Rabbits were able to produce specific IgG antibodies recognizing the respective immunization venom; even an in vitro interspecies cross-recognition was detected. The separation and purification processes allowed us to obtain IgG products without considerable contaminants (except for albumin). The process was characterized, and critical stages were identified.

## 1. Introduction

The World Health Organization (WHO) strongly recommends, in its antivenom production guidelines, that antivenoms must be produced at the requested needs of each country, with the venoms of their own medically important venomous animals, based fundamentally on the fact that cross-reactions are not always observed between venoms from different regions, making antivenoms lose their therapeutic efficacy and, therefore, their production in foreign countries is ineffective and not recommended [1]. According to this, different studies indicate that the available antivenoms in Latin America covering *Tityus* envenomation are not effective outside the country of manufacturing [2]. Currently, Colombia does not have any local production of antivenoms covering scorpion stings, although Colombia possesses an important diversity of medically important scorpions, with six different Class I–Class III species (according to Ward et al. [3]) classified in the epidemiological and medically important family of Buthidae (genus *Tityus* and *Centruroides*) [3]. The harmful scorpion fauna considered epidemiologically and medically important in Colombia correspond to species *Tityus pachyurus*, *Tityus asthenes*, *Tityus fuhrmanii*, and *Centruroides* spp. distributed in the central, north-west and north areas [3,4,5]. The only available therapy to treat scorpion stings in Colombia corresponds to an antivenom manufactured in Mexico, which is a third-generation polyclonal horse-derived formulation produced with the hyperimmune plasma of *Centruroides* individuals from Mexico [2].

In addition to the lack of a local antivenom producer, Colombia does not have any official active epidemiological program tracking scorpion stings, and the national incidence is unknown. Epidemiological data collected from two localities in Colombia indicate that the incidence of scorpion stings in Colombia may be estimated to be 4.5 per 100,000 inhabitants/year [6].

Although Buthidae scorpion venom has not been deeply characterized, previous studies reported their LD_50_, their biochemical profile, and protein content, indicating that these scorpions are an important source of peptides affecting mammal ionic channels and peptides with antimicrobial activity [4,5,7,8,9,10]. Their envenoming symptoms cover local and systemic signs, including local pain, tachycardia, vomiting, generalized sweating, drowsiness, abdominal pain, tachypnea, and sialorrhea, among others [5,6,11].

The lack of local antivenom production, despite the recommendation of the WHO, and the presence of harmful scorpion fauna from the Buthidae family, allowed us to propose the objective of this study focused on the characterization of a lab-scale process to produce the first antivenom specific for Colombia, covering scorpion stings produced by *Tityus pachyurus*, *Tityus asthenes*, *Tityus fuhrmanii*, and *Centruroides* spp.

## 2. Results

### 2.1. Characterization of the Antigen Batches

*Tityus pachyurus* venoms from Tolima were pooled as batch number V.T.P.; *Tityus asthenes* venoms from the Aburrá Valley, North, and Urabá sub-regions (Antioquia) were pooled as batch number V.T.A; *Tityus fuhrmanni* venoms from the Aburrá Valley and South-west sub-regions (Antioquia) were pooled as batch number V.T.F.; and *Centruroides* spp. venoms from the Aburrá Valley, Urabá, South-west and West sub-regions (Antioquia) were pooled as batch number V.C.E.

**Figure 1:**
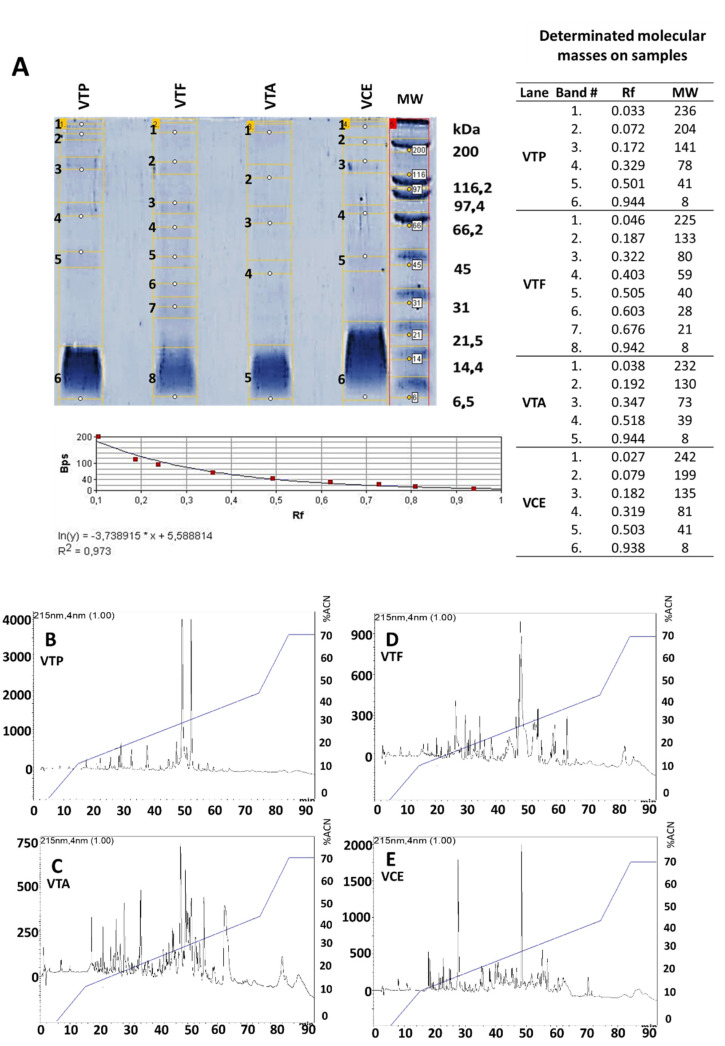
TRIS-TRICINE electrophoresis and chromatographic profile of each batch venom. (**A**) Ten percent TRIS-TRICINE gels loading 1.5 mg/mL of each venom batch. M.W.: Molecular weight. Calibration curve using a simple exponential fit approximation to determine molecular masses on each venom batch. Yellow lines correspond to samples while red line correspond to molecular weight markers. (**B**–**E**): RP-HPLC chromatographic profiles of the crude venom of all scorpions using a C18 column (250 mm–4.6 mm). Elution gradient used: 0–70% of acetonitrile (99% in TFA 0.1%). The run was monitored at 215 nm. (**B**): *Tityus pachyurus* venom batch V.T.P. (**C**): *Tityus asthenes* venom batch VTA. (**D**): *Tityus fuhrmanni* venom batch VTF. (**E**): *Centruroides* spp. venom batch V.C.E.

Figure 1 shows the TRIS-TRICINE electrophoretic and the RP-HPLC chromatographic profile of each venom batch from *Tityus pachyurus*, *Tityus asthenes*, *Tityus fuhrmanii,* and *Centruroides* spp. Molecular mass determination using a simple exponential fit approximation curve with an R^2^ of 0.973 showed antigen batches (venoms) with a molecular mass ranging from 8 kDa to 242 kDa, with the majoritarian compounds resolved among 8 kDa and 14.4 kDa (Figure 1A). The chromatographic profile shows a complex venom composition with major compounds eluting around 30% of A.C.N. (Figure 1B–E). In all cases, each venom batch showed a specific profile.

The Tityus pachyurus, *Tityus asthenes*, *Tityus fuhrmanii*, and *Centruroides* spp. venom batches LD_50_ values used in this study indicate that *Tityus pachyurus* is the most toxic venom of all four, with the lower LD_50_ value, while our *Centruroides* spp. mixture has a higher LD_50_ value (see Table 1).

### 2.2. Characterization of the Hyperimmune Plasma

**Figure 2:**
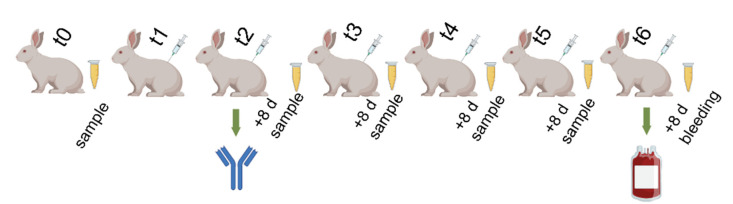
Immunization timeline. Rabbit immunization period using different antigen mixture amounts according to each LD_50_. Samples to determine antibody titers were collected 8 days after the immunization (+8 d) on t0, t2, t3, t4, t5, and t6; t0 was used as baseline or control. Bleeding was performed on t6.

Figure 2 shows the timeline of immunization and sample collection using t0 as the baseline or control. According to Figure 2, after t2, samples were collected to determine antibody titers (IgG). After final bleeding, we obtained an average of 56% of plasma from all rabbits (see Table 2). We detected no toxicological effect after the immunization process in any of the cases.

**Figure 3:**
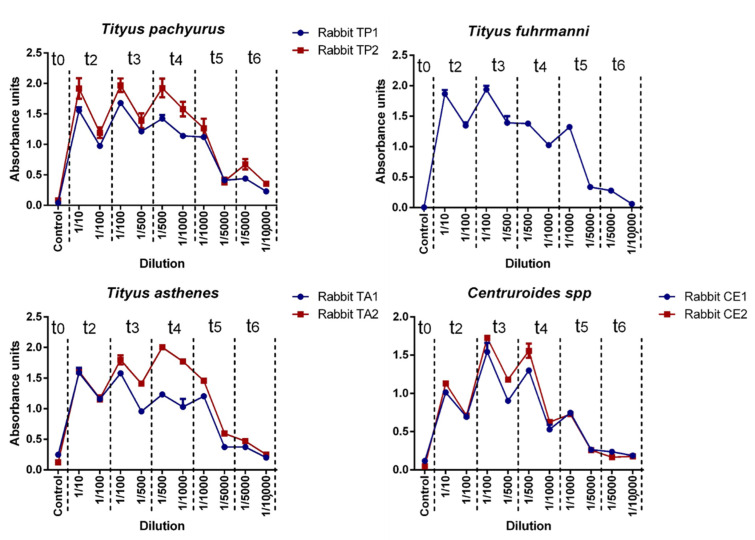
IgG quantification. Antibody production by antigen batch group after each immunization period. Only one rabbit was used on *Tityus fuhrmanni* venom.

Figure 3 shows the antibody titers by antigen batch group, expressed as the dilution rate able to be detected in each rabbit group. In all antigen batch groups, the maximum dilution detected was 1/10,000 on t6. Antibody titers varied between each rabbit immunized group but remained stable each time. All titers increased after the first antigen injection and maintained a constant production until t6, and their detection was possible in a dilution of 1:10,000. Despite the difference in some individuals of *T. pachyurus* and *Centruroides* spp. groups, IgG production in everyone was constant in the time period. *Tityus asthenes* individuals showed a considerable difference in IgG production after t3 at a 1:500 dilution.

**Figure 4:**
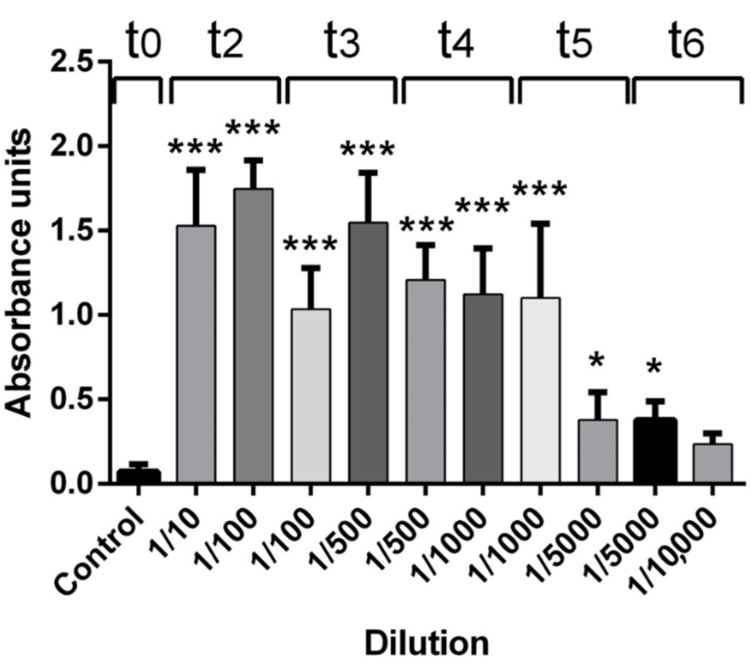
IgG quantification after pooling the absorbance of dilutions from all rabbits. Antibody production in all immunized rabbits by dilution after each immunization period after pooling absorbance of each dilution. Three asterisks indicate a significant difference with a *p* < 0.001 compared with the control group (t0). One asterisk indicates a significant difference with a *p* < 0.05 compared to the control group (t0). Absorbance recorded at 450 nm.

After pooling the absorbance of dilutions 1/10 to 1/1000 of the t2–t5 immunization periods from all rabbits, the IgG levels showed a statistically significant difference when compared with t0 (control, *p* < 0.0001), while dilutions 1/5000 from the t5–t6 immunization periods showed a statistically significant difference with *p* < 0.05 when compared with t0 (control) (see Figure 4). Despite the visible increment in the antibody titers on dilution 1/10,000 (t6), the results did not show a statistically significant difference compared with t0 (control).

**Figure 5:**
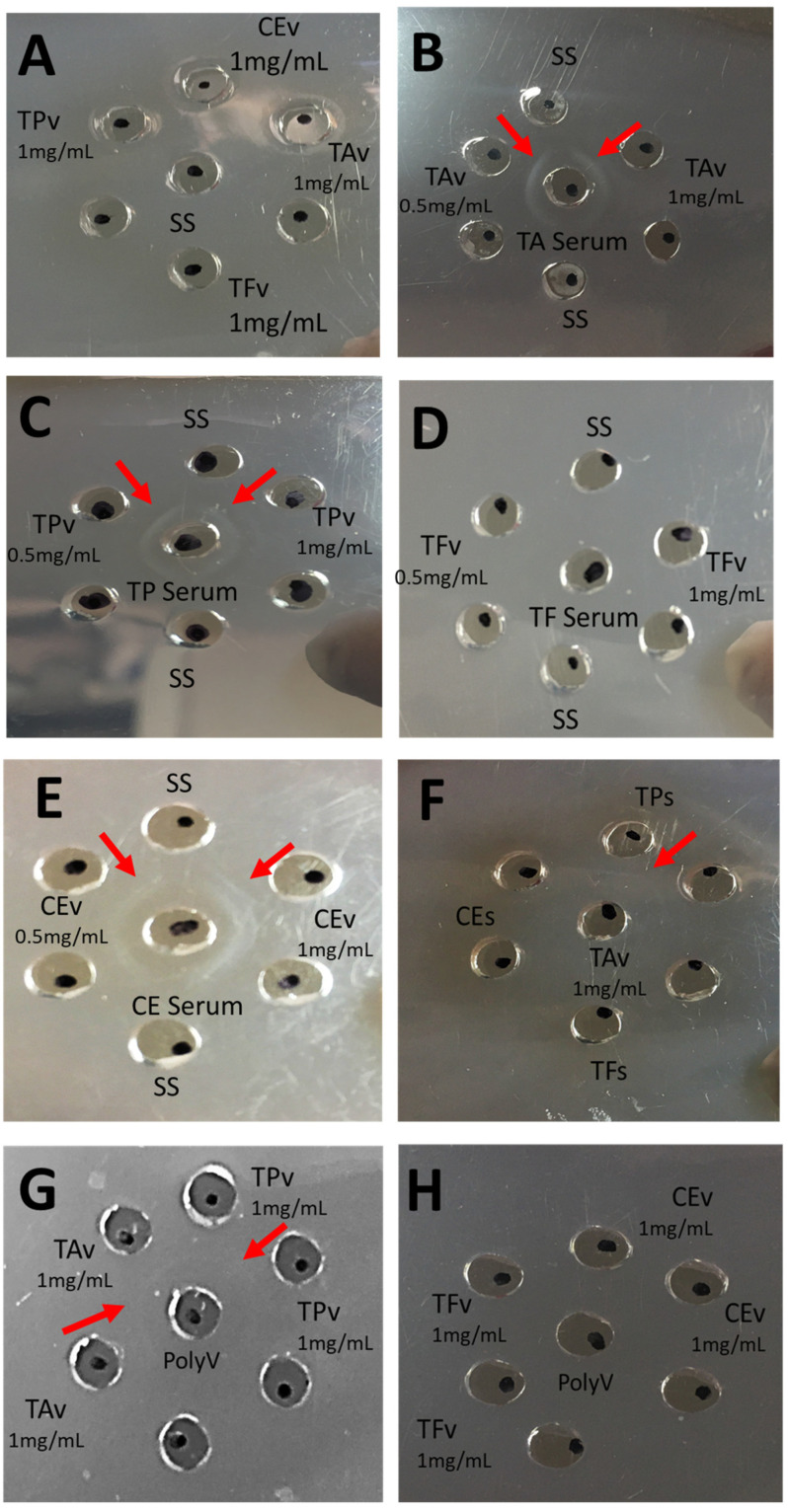
Double immunodiffusion assay. Antigen/antibody recognition assay of antigen batches (0.5 mg/mL and 1 mg/mL) against hyperimmune serums. (**A**): Negative Control using 0.9% saline physiological solution (S.S.); (**B**): *Tityus asthenes*; (**C**): *Tityus pachyururs*; (**D**): *Tityus fuhrmanni*; (**E**): *Centruroides* spp. (**F**): recognition of *Tityus asthenes* venom by different hyperimmune serums. (**G**,**H**): Recognition of pooled hyperimmune serum (PolyV) with different venoms (1 mg/mL). Arrows indicate venom and hyperimmune recognition. Red arrows indicate recognition sites.

For t6, the antigen/antibody reactivity analysis of the hyperimmune serum was able to detect an antigen concentration of 1 mg/mL and 0.5 mg/mL on *T. pachyurus*, *T. asthenes*, and *Centruroides* spp.; *T. fuhrmanni* was not recognized in any of the evaluated doses (see Figure 5B–E). The negative control did not show any reaction (see Figure 5A). In the antigen cross-reactivity analysis, *T. asthenes* venom (1 mg/mL) was recognized by *T. pachyurus* hyperimmune serum (see Figure 5F). After pooling all hyperimmune serum from all the rabbits (equimolarly), the pool was able to recognize venoms (1 mg/mL) from *T. pachyurus* and *T. asthenes*. In contrast, the venoms from *T. fuhrmanni* and *Centruroides* were not recognized at the evaluated doses (see Figure 5G,H).

### 2.3. Characterization of the Produced Antibodies and IgG Preparation

**Figure 6:**
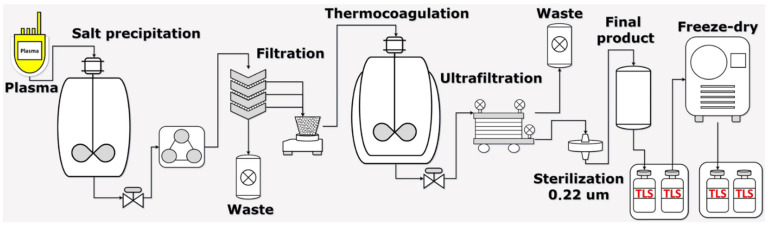
IgG purification scheme. The purification scheme of IgG is composed of two precipitation stages with ammonium sulfate. Three different kinds of filtration were used to purify the IgG, nominal, tangential (diafiltration and concentration), and sterile filtration.

After bleeding on t6 with an antibody titer of 1:10,000 on each rabbit, obtained plasma (by decantation) was pooled by antigen batch group. IgGs were purified from the plasma protein complex mixture following the purification scheme in Figure 6. In all cases, after processing each batch (separately), we were able to purify immunoglobulins from each hyperimmune plasma.

**Figure 7:**
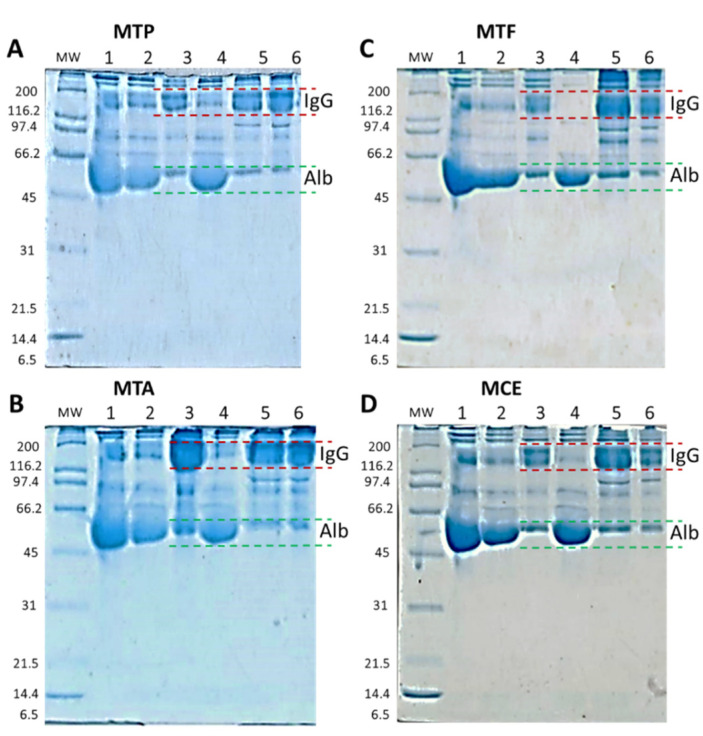
Monovalent batches IgG purification. Purification of IgG from hyperimmune plasma from (**A**): *Tityus pachyurus* monovalent batch (MTP); (**B**): *Tityus asthenes* monovalent batch (M.T.A.); (**C**): *Tityus fuhrmanni* monovalent batch (M.T.F.); (**D**): *Centruroides* spp. monovalent batch (M.C.E.). 1: Plasma pool. 2: Plasma before precipitation. 3: Precipitate (Biomass). 4: Filtrate. 5: Thermocoagulated before filtration. 6: Thermocoagulate precipitate. M.W.: Molecular weight. IgG: Immunoglobulins. Alb: Albumin.

**Figure 8:**
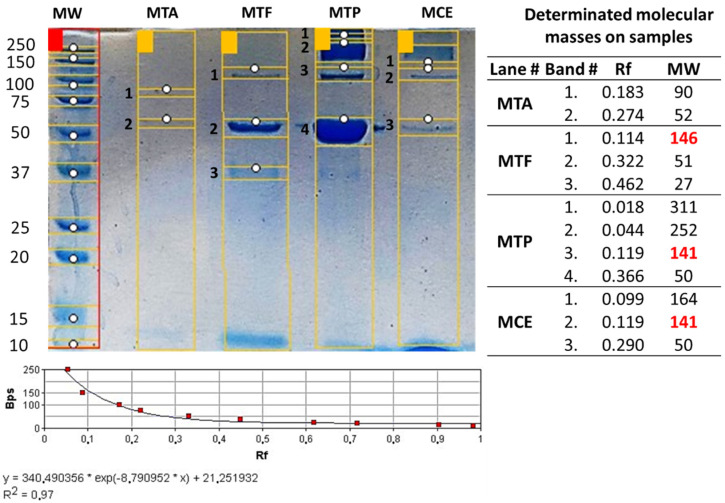
Monovalent batches electrophoresis. Ten percent TRIS-TRICINE gels loading 1.5 mg/mL of purified IgG after tangential flow filtration (final product). M.W.: Molecular weight. Calibration curve using an exponential fit approximation to determine molecular masses on each purified IgG batch. Red highlighted molecular weights indicate bands matching molecular masses of IgG. M.T.A.: *Tityus asthenes* monovalent batch; M.T.F.: *Tityus fuhrmanni* monovalent batch; MTP: *Tityus pachyurus* monovalent batch; M.C.E.: *Centruroides* spp. monovalent batch.

Table 3 shows the protein and albumin performance during the purification process to obtain the purified IgG. Rabbit plasma showed a protein concentration average of 82.47 ± 20.37 mg/mL, and an average of 38.08 ± 1.05 mg/mL of albumin, corresponding to 46% of the total protein content. After the precipitation process with a saturated solution of (NH_4_)_2_SO_4_ and the filtration, the recovered biomass (see Figure 7A–D, line 3) showed a higher concentration of IgG compared with the filtrate, where the presence of albumin is more evident (see Figure 7A–D, line 4). This process allowed the elimination of more than 87.9% ± 3.9% of the total albumin present (plasma before precipitation/Precipitate-Biomass—see Table 3). The thermocoagulation process allowed the precipitation of different proteins (including probable immunoglobulins), leaving a complex IgG solution (filtrate) with albumin present (see Figure 7A–D, lines 5 and 6). After the tangential flow filtration process, we obtained a clear and purified IgG with the presence of albumin (not perceptible in the TAM170219 batch) (see Figure 8).

By subtracting the albumin concentration from the protein concentration at the end of the process (Diafiltrade FP), we were able to recover 6.4% ± 3.9% of specific IgGs from the total amount of proteins present in the plasma before the precipitation stage (see Table 3) (1.3% *T. asthenes*; 9.7% *T. pachyurus*; 9.3% *T. fuhrmanni* and 5.4% *Centruroides* spp.); while the albumin levels decrease to levels below 1% (0.9% ± 0.3%) at the end of the process (Diafiltrade FP) from the total amount of albumin present in the plasma before the precipitation stage (0.8% *T. asthenes*; 1.2% *T. pachyurus*; 0.5% *T. fuhrmanni* and 1.3% *Centruroides* spp.). Despite the representative decrease in the total albumin from the plasma, the final product showed a 10.3% ± 7.7% average albumin content, with the highest relation being the *T. asthenes* monovalent batch (21%) and the highest concentration for *T. pachyurus* (0.3 mg/mL).

### 2.4. Monovalent IgG Efficacy

The preliminary in vivo efficacy test showed effectivity in the *Tityus asthenes* monovalent batch (M.T.A.) at a concentration of 50 mg/mL against 3 LD_50_ (0.06 LD_50_/mg of rabbit IgGs). At this concentration, we were able to detect a prolongation in the expected lifetime above 2 h and to reduce the symptomatology of *T. pachyurus* venom (pain, salivation, piloerection, hyperventilation, distress, and excitability) in the control group.

## 3. Discussion

Colombia is one of the most diverse countries for arachnids worldwide, housing around 81 different scorpion species, distributed in 14 different genera [12]. Despite the presence of these vast number of species, including Buthidae individuals from the *Centruroides* and *Tityus* genus (causing severe accidents), Colombia does not have a program to produce antivenoms covering scorpion accidents [2].

Venoms from Buthidae scorpions from Colombia are a complex mixture of proteins with a molecular mass ranging from 6 kDa up to 210 kDa [9]. The electrophoretic and chromatographic profiles from all four venoms analyzed in this study showed similar patterns to the previous descriptions of these venoms from Colombia, including the molecular mass ranges and the venom complexity (chromatographic profiles) using similar conditions [5,8,9]. *T. pachyurus* LD_50_ value (53.3 µg/mice or 2.6 mg/kg) showed a lower LD_50_ value against the other venoms in concordance with the result published by Otero et al. and Barona et al. [4,6]. The LD_50_ values from *T. pachyurus*, *T. fuhrmanni*, and *Centruroides*, were similar to previous LD_50_ reports (although below) by Mendoza-Tobar et al., Gomez et al., and Estrada-Gomez et al., respectively [5,13,14]. Only *T. asthenes* venom showed a difference from the previous report by Gomez et al. [13]. With the *Centruroides* venom batch, we could not calculate the LD_50_ value using concentrations up to 25.0 mg/kg, detecting at this concentration only toxic effects characterized by pain, salivation, piloerection, hyperventilation, distress, and excitability. Estrada-Gomez et al. previously described a similar result in the species *C. edwardsii* from the Antioquia and Tolima provinces using concentrations of 19.2 mg/kg [5]. In the same species, Brenes and Gomez established an LD_50_ of *C. edwardsii* from Costa Rica at 4.4 mg/kg [15]. In a different *Centruroides* species from Colombia, Mendoza-Tobar et al. calculated an LD_50_ of 19.5 mg/kg in *C. margaritatus* from the Cauca province. The LD_50_ discrepancies with previous reports for *T. asthenes* and *Centruroides* may be due to individual geographical variability and the interspecific differences as previously reported in the venom from *C. edwardsii* and *T. pachyurus* from Colombia [5,16]. In the case of *T. asthenes*, individuals used by Gomez et al. were collected exclusively in the Uraba sub-regions (Antioquia). In contrast, we used individuals from the Aburra Valley, North and Uraba sub-regions (Antioquia). In the case of *Centruroides*, when comparing our result with Mendoza-Tobar, they used specimens from a different geographical location (Cauca province), while we used individuals from the Antioquia province where the main *Centruroides* species distributed are *C. edwardsii* [5,17] and *C. margaritatus* [17].

There are no previous scientific reports of antibodies against scorpion venom production in Colombia. The presence of antibodies against each venom batch (antigens) was tested by ELISA assay. All rabbits were able to produce IgG according to the respective stimuli used (venom), with titers ranging from 1 × 10^1^ U-ELISA/mL and 1 × 10^4^ U-ELISA/mL. Anti-scorpion IgG antibody production in rabbits performed a similar time-dependent IgG production (in different magnitudes) and constant IgG production in each individual (in the same rank), as previously reported by Guidolin et al. in the production of equine IgG against snake venoms [18,19]. Although we observed statistically significant differences in IgG production in each rabbit group, the general production behavior was similar. Ouchterlony indicates that the rabbit IgGs produced showed specificity (recognition) against the respective immunization venoms. In the case of *T. asthenes*, hyperimmune serum showed cross-recognition against *T. pachyurus* venom, as reported by Borges et al. [2]. Scorpion venoms from the same genera share similitude in their venom content, especially in the *Tityus* genus; as previously reported by Estrada *et al*., where *T. asthenes* venom showed similarity with *T. serrulatus*, *T. bahuiensis*, *T. discrepans* and *T. pachyurus* [9]. The IgG production could be improved by using higher venom concentrations to reach titers over 1 × 10^4^ U-ELISA/mL before final bleeding. To do so, it is very important to consider that we did not detect any toxic effect at the venom doses used on each rabbit (we used doses 9.2 times for *T. pachyurus*, 12 times for *T. asthenes,* and 11.4 times for *T. fuhrmanni* below their respective LD_50_).

The specificity of the antibodies produced and the approach of the IgG purification process in monovalent batches allowed the further purification of specific IgG with slight albumin contamination. In all cases, we recovered an average of 6.9% ± 3.9% of protein, and after subtracting the concentration of albumin detected, the total amount of protein corresponds mainly to total IgG (6.4% ± 3.9%). Electrophoretic profiles corroborate this result since, in the final product, we detected bands with molecular masses similar to IgG and albumin. The *T. asthenes* batch (M.T.A.) showed lower values of recuperated proteins at the end of the process, with strong losses after the precipitation and thermocoagulation processes. However, the volume of processed plasma and IgG titers was similar to the other groups. The most significant loss is observed in the thermocoagulation process just before the filtration, indicating a possible loss of IgG by precipitation. Figure 7B, line 6, shows this is likely lost with the high concentration of proteins with a molecular mass similar to IgG in the thermocoagulation precipitate. At the end of the M.T.A. batch, we were able to obtain lower values of total protein concentration—1.7% of the total protein—with albumin corresponding to 21% on the protein measure. The precipitation with (NH_4_)^2^SO_4_ allowed a deep purification of IgG, precipitating 98% ± 3.7% of the total albumin present in the plasma. This is an exceptionally important characteristic in antivenoms since albumin is the main contaminant in final products and its presence compromises the safety of the product since it is responsible for early adverse reactions after antivenom administration [1]. Despite this important decrease in total albumin, we were not able to reach the WHO standards, which indicates that albumin concentration should not exceed 1% of total protein content [1]. To obtain the requested concentrations suggested by the WHO, further improvements could be performed in the T.F.F. step to diminish the concentration of albumin in the final product, adding a specific polyethersulfone albumin cassette just after the hydrosart filtration.

Although the purified IgG anti-*T. pachyurus* was able to neutralize the toxic effects, diminish symptoms, and prolong mice lifetime, the venom/IgG ratio (0.3 LD_50_/1 mg) used in this research was low compared with commercial antivenoms, which can neutralize 0.3 LD_50_/1 mg of *Centruroides* venoms from North America or *T. pachyurus* venom from Colombia [4,20]. Further studies are required to test higher concentrations of IgG. Obtaining higher amounts of IgG to be processed could be achieved by obtaining plasma with higher titers of IgG. To raise the IgG titers on rabbits, they can be immunized with higher concentrations of venoms since we used doses 9.2 times, 12 times and11.4 times below the respective LD_50_ for *T. pachyurus*, *T. asthenes,* and *T. fuhrmanni*, respectively. The fractionation method is efficient in separating and purifying the specific IgG. Processing hyperimmune plasmas with higher titers of IgG should allow the purification of higher concentrations of IgG in the final product.

## 4. Materials and Methods

### 4.1. Venoms and Antigen Preparation

Scorpions with epidemiologic and clinical importance in Colombia, according to [3,6,11], were selected for this research and included *Tityus pachyurus*, *Tityus asthenes*, *Tityus* *fuhrmanni*, and *Centruroides* spp., collected in the provinces of Antioquia and Tolima, and kept in captivity in the serpentarium of the University of Antioquia (COLBIOFAR-149) with water ad libitum and fed with insects (*Periplaneta americana* and *Tenebrio molitor*). Tityus pachyurus venom from Tolima, including 31 individuals was pooled in the *T. pachyurus* batch; *Tityus asthenes* venom from the Aburra Valley, North, and Urabá sub-regions (Antioquia) included 27 individuals; *Tityus fuhrmanni* venom from the Aburrá Valley and South-west sub-regions (Antioquia) included 140 individuals and *Centruroides* spp. venom from the Aburrá Valley, Urabá, South-west and West sub-regions (Antioquia) included 67 individuals, females, and males in all cases. Each antigen mixture was identified with a batch number composed of the letter V of “venom” and the first genus letter followed by a second letter corresponding to the scorpion species. Each antigen batch preparation was characterized using protein quantification, electrophoresis, rp-HPLC, and LD_50_, as described below.

### 4.2. Electrophoretic Profile

Antigens batches (venoms) were analyzed using 10% TRIS-TRICINE gels and stained with Coomassie blue R-250, and molecular weights were estimated using standard broad range markers (unstained SDS-PAGE standards broad range # 1,610,317 Bio-Rad, Hercules, CA, USA). Quantification of volumes and calculation of molecular weights were performed using the software GelAnalyzer 19.1, available at: http://www.gelanalyzer.com/ (accessed on 2 February 2022) [21]. Molecular weights were calculated using the known values of the standard broad rank markers (Bio-Rad): 200 kDa, 116 kDa, 97 kDa, 66 kDa, 45 kDa, 31 kDa, 21 kDa, 14 kDa, 6 kDa. To estimate the molecular weight, we used a simple exponential fit approximation of the Rf (retention factor, measured as the band distance migrated/gel length) of each analyzed band. Samples were loaded at a concentration of 1.5 mg/mL and a final volume of 20 µL. Each antibody purification/preparation stage and antivenoms batches were analyzed using 12% sodium dodecyl sulfate-polyacrylamide gels (SDS-PAGE) according to Laemmli [22] and stained with Coomassie blue R-250. Molecular weights were estimated using standard broad range standards (Bio-Rad). Samples were loaded at a concentration of 1.5 mg/mL.

### 4.3. Chromatographic Profile

Antigens batches (venoms) profiles were obtained using reverse-phase high-pressure liquid chromatography (RP-HPLC) using a Shimadzu Prominence chromatograph LC-20AT (pump unit). One mg of crude venom was dissolved in 200 μL of solution A (0.1% TFA in water) and centrifuged at 3500× *g* for 5 min at room temperature. The supernatant was fractionated using a C18 RP-HPLC analytical column (250 × 4.6 mm), equilibrated, and eluted at a flow rate of 1.0 mL/min first isocratically (5% B for 5 min), followed by a linear gradient of 5–15% B for 10 min, 15–45% B for 60 min, and 45–70% B for 12 min according to with [9,23]. The chromatographic separation was monitored at 215 nm.

### 4.4. Median Lethal Dose

All animal experiments were carried out in Swiss-Webster mice (18–20 g body weight). The lethality induced by each scorpion venom was evaluated by injecting doses ranging from 25 µg up to 121 µg, all diluted in 250 µL of PBS and administrated by the intraperitoneal (i.p.) route, in groups of four mice. The control group received PBS alone. Deaths were recorded during 48 h, and the Spearman–Karmer method was used to estimate the median lethal dose. All procedures were in accordance with the guidelines of the Ethics Committee of Universidad de Antioquia.

### 4.5. Animals

Adult rabbits weighing between 3.0–4.0 kg were used to produce the antivenoms. They were divided into 4 groups: *T. pachyurus* group *n* = 2, *T. asthenes* group *n* = 2, *T. fuhrmanii* group *n* = 1, and *Centruroides* spp. group *n* = 2. Animals were housed at serpentarium bioterium of the Universidad de Antioquia with water and food *ad libitum*. Before the immunization process, all animals were housed for 40 days, submitted to a deparasitation, and included in a clinical and para-clinical surveillance program. All procedures followed the guidelines of the Universidad de Antioquia Ethics Committee.

### 4.6. Immunization and Bleeding

Polyclonal antibodies were obtained by the continuous s.c. injection, during 93 days, with injections every 21 days of the antigen mixture across the back area (around the lymphatic back area). Each rabbit group was immunized using 857 µg of *T. pachyurus* (Batch number: TP200219), *T. asthenes* (Batch number: TA200219), *T. fuhrmanii* (Batch number: TF200219), and *Centruroides* spp. (Batch number: CE200219). All antigens were diluted in Marco Montanide I.S.A. 50 (Brazil) and mixed with Tween 80 (complete MMT80) for injection. Rabbits were bled to collect exploratory samples in the central ear vein using dry tubes (red cap) to collect serum samples for antibodies quantification every eight days after each antigen injection for two months (t0, t1, t2, t3, and t4–t0 was used as a baseline or negative control). With the appropriate antibody concentration, rabbits were anesthetized using ketamine and xylazine (1:1) and bled by intracardiac punction using a 20 Gauge × 1 1/2 inch needle connected to a 20 mL syringe containing an anticoagulant solution. Plasma and cells were separated by centrifugation using 500× *g* (2000 revolutions per minute) for 15 min at room temperature. The collected plasma was measured and stored at 2–8 °C until use.

### 4.7. Antibodies Quantification

Rabbit IgG titers were determined by enzyme-linked immunosorbent assay (ELISA), according to [24], with some modifications. Plates (Corning Costar microplates ref 3591) were coated overnight, at 4 °C, with 100 µL/well of the respective antivenom batch antigen (4000 ng/mL in Carbonate/Bicarbonate Buffer, pH 9.6). After a washing step, the remaining binding sites were blocked with coated buffer containing 2% B.S.A. (Bovine Serum albumin, Low Heavy Metals US112659-100GM Albumin, Calbiochem) for two h at 37 °C, followed by a washing step. Rabbit hyperimmune serum was diluted in sample buffer (PBS pH 7.4, containing 1% B.S.A.) in concentrations from 1:10 to 1:10,000 (serial dissolutions). Then 100 µL of commercial antivenom dilution or similar preparation of immunoglobulins from a non-immunized rabbit (as controls) were added to the respective well according to the antigen, incubated for two h at 37 °C, and washed. IgG anti-rabbit and IgG- rabbit radish peroxidase conjugate diluted 1:8000 in sample buffer, was added (100 µL/well), incubated for 2 h at 37 °C, and washed. Finally, 100 µL O.P.D. -ortho-phenylenediamine (Amresco) diluted at 0.1% in citrate buffer, pH 6.0, containing 0.1% hydrogen peroxide, was added. Plates were protected from the light, incubated for 20 min at 37 °C, and absorbances were recorded using a Thermo Scientific Multiskan FC microplate reader at 450 nm.

### 4.8. Double Immunodiffusion

The immunological properties of each antigen were evaluated using the double immunodiffusion technique described by Ouchterlony [25]. Serum samples were collected, as explained above. Using acetate sheets covered with agarose 1%, antigens solution (20 µL) was placed in peripheral wells (antigen concentrations used were 0.5 mg/mL and 1 mg/mL), whereas the central well was filled with 20 µL of hyperimmune serum. The antibodies in the antiserum react with both the antigens resulting in a smooth line of precipitate.

### 4.9. Antibody Purification and IgG Process Characterization

IgG purification and fractionation were performed according to the process described by Guidlolin et al., with some modifications [18]. Each rabbit group plasma was purified and fractionated separately in monovalent batches according to the used antigen (venom). For this, a saturated solution of (NH_4_)_2_SO_4_ (Ammonium sulfate EMPROVE^®^ EXPERT—Merck, Darmstadt, Germany) was added to each pooled plasma obtained from the rabbits immunized with the respective scorpion venoms. After 2 h of stirring, the mixture mainly containing immunoglobulins was separated by filtration using 10 µm pore size nitrocellulose membranes (Ahlstrom Munktell Quantitative Filter Paper, Sheets, Grade 1602N). Membrane cake was re-suspended, and the pH was adjusted to a neutral value with a low protein donor acid. Non-IgG proteins were precipitated by adding a second round of a saturated solution of (NH_4_)_2_SO_4_ (Ammonium sulfate EMPROVE^®^ EXPERT—Merck) and incubated at 55 °C for 1 h under stirring. The mixture was filtered again using 10 µm pore size nitrocellulose membranes (Ahlstrom Munktell Quantitative Filter Paper, Sheets, Grade 1602N), and the membrane cake was discarded. The permeate was subsequently filtrated using a tangential flow filtration (T.F.F.) process using a ready-to-use and reusable laboratory crossflow tangential cassette with a polyethersulfone membrane (Vivaflow 50R—Sartorius) to remove salts and concentrate the resultant IgG antibodies in the same equipment. Each monovalent batch was placed at 4 °C until use with a batch number assigned (see below). In the end, the resultant IgG solution was sterilized by passing through a 0.22 µm filter membrane and freeze-dried using a Labconco Lyph-lock 6 L, Model 77,535 (Labconco Corporación, Kansas City, MO, USA). The freeze-dry conditions included a temperature delta from −41 °C to 15 °C and a pressure of 50 × 10^−3^ mBar. Final products and each production process stage of each monovalent batch were analyzed to confirm the presence of IgG by comparing their relative molecular weight with standard molecular weight markers using gel electrophoresis and the software GelAnalyzer 19.1. Total protein concentration and albumin concentration were also quantified at each production process stage, including final products in each monovalent batch. Each monovalent batch was identified with a batch number composed of the letter M (indicating a monovalent batch) and the first genus letter followed by a second letter corresponding to the scorpion species.

### 4.10. Protein Quantification

Protein concentration of each production process stage, including final products in monovalent batches, was determined, following the Biuret method using Bio-Rad Protein Assay reagent and B.S.A. (Biosystems ref 11698) as standard [26,27,28]. Briefly, 750 µL of Biuret reactive (MOL LABS, Bogotá, Colombia) were added to 250 µL of 1:20 dilution of each sample. The mixtures were incubated for 30 min at 37 °C, and the absorbance was measured at 540 nm. Additionally, a calibration curve was created with different albumin concentrations ranging from 0.1 mg/mL to 50 mg/mL, working under the conditions previously described (y = 0.0592X + 0.0204—R^2^ = 0.9944). Once the sample was read, the value was interpolated in the calibration curve to measure the albumin quantity.

### 4.11. Albumin Quantification

Albumin concentration of each production process stage, including final products in monovalent batches, was determined following the method described by Doumas et al. [27] using a specific commercial kit for albumin determination (Biosystems Albumin Reagent with bromocresol green ref 11547). Briefly, 10 µL samples were mixed with 1000 µL of albumin reactive. The mixtures were mixed using a vortex, and the absorbance was measured at 630 nm. Additionally, a calibration curve was created with different albumin (BSA) concentrations ranging from 5 mg/mL to 50 mg/mL, working under the conditions previously described (y = 0.0068X + 0.001—R^2^ = 0.9978). Once the sample was read, the value was interpolated in the calibration curve to measure the albumin quantity.

### 4.12. Efficacy Test

A preliminary efficacy test was performed to test the ability of the specific IgG to neutralize the venom toxic effects. From all monovalent batches, the batch antivenom from *Tityus pachyurus* was tested since this venom presented the lowest LD_50_ among all other venoms. Different amounts of *Tityus pachyurus* of monovalent batches (1 mg, 5 mg, and 10 mg) were tested against 3 LD_50_. Mixtures of antivenom and venom were incubated at 37 °C for 30 min. After incubation, the mixture was injected intraperitoneally into Swiss mice weighing 18–20 g. One control group was injected only with the respective 3 LD_50_.

### 4.13. Ethical Statement

All animals used in this study were maintained and treated under strict ethical conditions following the WHO Guidelines for the Production, Control, and Regulation of Snake Antivenom Immunoglobulins [1]. All procedures involving animals were carried out with the support of the veterinaries Laura lopez Diez, Angélica Zuluaga Cabrera PhD and Javier Murillo PhDc. Protocols were approved by the Ethics Committee of Animal Usage in Research (CEEA, University of Antioquia—Rectoral Resolution 18,084) Minute No.: 123-2019.

### 4.14. Statistical Analysis

Results were expressed as mean ± standard error media (S.E.M.), and statistical comparisons were made using a one-way ANOVA with a Dunnet’s post-test when comparing the median of each group or a Tukey’s post-test when comparing the median of each group with the media control group. In all cases, a difference statistically significant was assumed when *p* < 0.05. All data analysis was carried out using GraphPad PRISM 5 (GraphPad Software, Inc.; La Jolla, CA, USA).

## 5. Conclusions

The immunization and the fractionation processes allowed the production of rabbits’ hyperimmune plasma that was subsequently fractionated and purified into IgG antibodies specific against *Tityus pachyurus*, *Tityus asthenes*, *Tityus fuhrmanii*, and *Centruroides* spp. venom. The plasma fractionation method is definitely adequate to separate and purify the specific IgG. The obtained results could be enhanced by improving the immunization process using a higher concentration of venom, while the production process could be improved to reduce albumin levels and increase the concentrations of the final protein. These improvements can be used to obtain further batches with a higher concentration of IgG, allowing a formulation to achieve a polyvalent antivenom covering all venoms.

## Figures and Tables

**Table 1:** Scorpions venoms LD_50_. Median lethal doses (LD_50_) of each scorpion venom batch, determined using intraperitoneal (I.P.) injection route, with 95% confidence intervals and calculated by non-linear regression. L.D.: Lethal dose.

Scorpion Species	*Tityus pachyurus*	*Tityus asthenes*	*Tityus fuhrmanni*	*Centruroides* spp.
**Antigen batch**	VTP	VTA	VTF	VCE
**IP LD_50_ µg/mouse**	53.3 (32.6–150)	68.83 (35.7–185.7)	64.1 (40.3–246)	>500

**Table 2:** Collected blood volume and plasma quantification.

	Blood Volume (mL)	Plasma Volume (mL)	Plasma Percentage
***Tityus pachyurus***	35	21	60%
	37.7	19	50%
***Tityus asthenes***	37.5	22.5	60%
	35	17.5	50%
***Tityus fuhrmanni***	36	21	58%
***Centruroides* spp.**	40	22.5	56%
	25	15	60%

**Table 3:** Protein and albumin percentages on the different IgG purification stages from each monovalent antigen batch. F.P.: Final Product. M.T.A.: *Tityus asthenes* monovalent batch; M.T.F.: *Tityus fuhrmanni* monovalent batch; MTP: *Tityus pachyurus* monovalent batch; M.C.E.: *Centruroides* spp. monovalent batch. Light gray shadow lines indicate the waste product of the process.

IgG Process Purification Stage	MTA		MTP		MTF		MCE	
	Protein	Albumin	Protein	Albumin	Protein	Albumin	Protein	Albumin
	%	%	%	%	%	%	%	%
Pool Plasma	100%	100%	100%	100%	100%	100%	100%	100%
Plasma before precipitation	65%	64%	65%	64%	48%	48%	58%	59%
Precipitate (Biomass)	6%	7%	10%	6%	13%	9%	19%	6%
Filtrate Waste	61%	61%	58%	67%	33%	48%	51%	60%
Termocoagulated before filtration	10%	11%	14%	20%	62%	46%	32%	24%
Diafiltrade Waste	0%	1%	0%	1%	7%	3%	4%	0%
Diafiltrade FP	1.10%	0.50%	7%	1%	5%	0%	3%	1%

## Data Availability

The data presented in this study are available in article.
